# Evaluation of Biophysical Properties of Potential Materials for the Manufacture of Protective Garments for Preterm Infants

**DOI:** 10.3390/ma15144878

**Published:** 2022-07-13

**Authors:** Karolina Wilgocka, Ewa Skrzetuska, Izabella Krucińska, Witold Sujka

**Affiliations:** 1 Institute of Material Science of Textiles and Polymer Composites, Faculty of Material Technologies and Textile Design, Lodz University of Technology, 116 Żeromskiego Street, 90-924 Lodz, Poland; ewa.skrzetuska@p.lodz.pl (E.S.); izabella.krucinska@p.lodz.pl (I.K.); 2 Tricomed SA, 5/9 Świętojańska Street, 93-493 Lodz, Poland; witold.sujka@tzmo-global.com

**Keywords:** protective garments, neonatal thermoregulation, thermal insulation, premature babies, biophysical comfort

## Abstract

Preterm infants, due to immature and dysfunctional skin, have increased water loss through the skin and consequently a decreased body temperature. In order to develop protective garments for preterm infants, it is important to select materials that will protect the child against water and heat loss. The authors are currently involved in the development of protective garments for premature babies, which are similar to baby clothes and contain a membrane that is partially permeable for vapor in combination with textile materials. This article presents the study of materials intended for the production of protective garments for pre-term infants. Samples of materials were investigated to determine biophysical comfort (tests of heat resistance, vapor resistance according to PN-EN ISO 11092:2014-11 and air permeability according to PN-EN ISO 9237) and porosity, surface mass in accordance with PN-EN 12127, and thickness in accordance with PN-EN ISO 5084. In order to determine the porosity of materials and to visualize the structure, tests on computer microtomography were carried out. The mechanical properties of the tested materials and the evaluation of the total hand value were characterized; the samples were tested on the KES device. The aim of this study was to select the most suitable fabrics for protective garments for premature infants to prevent excessive heat and moisture loss from the body, which can lead to hypothermia. For laminates, the optimal results of vapor resistance and heat resistance were obtained for laminate (15 g·m^−2^ PE foil + 15 g·m^−2^ PP non-woven), with a level of thermal resistance of 0.0766 m^2^·K·W^−1^ and vapor resistance of 188.729 m^2^·Pa·W^−1^, and for laminate (15 g·m^−2^ PE foil + 10 g·m^−2^ PP non-woven), with a level of thermal resistance of 0.0683 m^2^·K·W^−1^ and vapor resistance of 164.085 m^2^·Pa·W^−1^. For knitted fabrics, knitwear single cotton 155 g·m^−2^ showed the highest thermal resistance (0.0296 m^2^·K·W^−1^), and knitwear interlock polyester 120 g·m^−2^ showed the lowest thermal resistance (0.0179 m^2^·K·W^−1^). Knitwear cotton 120 g·m^−2^ had the highest water vapor resistance (8.402 m^2^·Pa·W^−1^), while knitwear interlock polyester 130 g·m^−2^ sample had the lowest resistance (6.356 m^2^·Pa·W^−1^). Garments for premature babies should have moisture barrier properties and high thermal insulation. They should also be characterized by optimal air permeability properties. Sample two-layer laminate (15 g·m^−2^ PE foil + 15 g·m^−2^ PP non-woven) had the best vapor resistance and thermal insulation properties. Moreover, this sample was characterized by good air permeability and surface weight compared to the other laminate samples. During the design of garments for premature babies, it is important to reduce the surface weight to as low as possible. Among the knitted fabrics, a knitwear single cotton 120 g·m^−2^ knitwear polyester interlock 120 g·m^−2^ was selected for having the best THV or tactile comfort. In addition, these knits were chosen for their lower surface weight. Based on the conducted tests, two-layer laminate (15 g·m^−2^ PE foil + 15 g·m^−2^ PP non-woven), the knitwear single cotton 120 g·m^−2,^ and knitwear polyester interlock 120 g·m^−2^ were selected for further research.

## 1. Introduction

Preterm infants, due to having immature skin, underdeveloped stratum corneum of the epidermis, and dysfunctions in its functioning, belong to the group of users of special purpose garments distinguished by specific biophysical parameters [1].

According to the World Health Organization definition, a preterm baby is an infant born after the 22nd week of gestation and before the 37th week of gestation. Extreme prematurity is defined as infants born before the 32nd week of gestation [2]. A major problem associated with preterm babies is high transepidermal water loss and related heat loss because of their immature skin, and the fact of having a large external surface area in relation to body weight [3]. The lack of stratum corneum and thinner epidermis in children born prematurely contribute to the loss of water through the skin, which also causes hypothermia. The immature skin of prematurely born children is characterized by higher susceptibility to damage as a result of even the smallest injuries [1,4].

The skin is the largest human organ and is responsible for creating a barrier between the body and the environment, so it protects the body from any mechanical injury, or chemical and biological stimuli. The skin plays a crucial role in fluid and temperature regulation and acts as an intermediary for the sensations of cold, heat, touch, pain, and comfort. The skin of premature infants born before 32 weeks gestation has an immature structure and insufficient function, so it should be observed and treated as an immature organ requiring special care and intervention. The neonatal skin consists of three main layers: the stratum corneum, the epidermis, and the dermis. The surface layer is the epidermis, while the dermis is a deeper layer formed of connective tissue. The stratum corneum forms at 21 weeks of gestation, and at 28 weeks the stratum corneum consists of two or three layers of cells. At 32 weeks, the baby has more than 15 layers, which is equivalent to adult skin. Premature infants born before 32 weeks have a very thin stratum corneum that does not sufficiently protect against transepidermal water loss, absorption of external factors, and microbial invasion. The magnitude of transepidermal loss is determined by body surface area, so in premature babies, increased body surface area to body weight ratio is an additional factor that increases transepidermal skin loss [5,6].

Water and heat loss in the premature infant depends on postnatal age and infant weight. Transepidermal water loss decreases with increasing postnatal age. Furthermore, low birth weight preterm infants lose about 150 mL of water per 1 kg of body weight within the first 24 h of life, whereas infants born after the 30th week of gestation lose about 12 mL per 1 kg of body weight. Such a condition is dangerous and may lead to dysfunction and electrolyte imbalance [3,7].

The transport of heat between the human body and the environment is a continuous process occurring mainly through the skin. Under normal conditions, humans lose about 80–90% of heat through the skin and about 10–20% through the respiratory tract. The mechanisms of heat loss can be divided into active and passive. Active heat exchange is the physical transfer of heat from a medium of higher temperature to a medium of lower temperature, while passive heat loss occurs by conduction, convection, or radiation. Heat loss by radiation, resulting from the temperature difference between the body and surrounding objects, is the main source of heat loss in the human body. During passive heat exchange, thermal energy may be transferred or absorbed, i.e., the body may be either cooled or overheated. During active heat exchange, however, heat is always transferred to the environment because it occurs only when the passive heat exchange is insufficient and sweat from the skin surface starts to evaporate; this phenomenon leads to the overheating of the body [7,8].

The expression thermal comfort is used to describe the state in which the body maintains thermal equilibrium. It is a state of human satisfaction with environmental conditions, i.e., one in which there is no need to lower or raise the temperature [9]. The phenomenon of thermal comfort is affected by various parameters characterizing the human body, the environment in which the person is located, and also the clothing which the person is wearing. Clothing ensures protection from the harmful effects of external factors and also serves to regulate heat between the body and the environment. Heat exchange is highly dependent on the thermal insulation of clothing, which consists of the thermal properties of the raw materials from which the clothing is made and the geometric structure of the materials [10,11,12].

Fabric solutions for premature babies are still being developed that maintain the right body temperature while wicking moisture away from the baby’s skin. Polymer materials, in comparison to cotton, have a lower thermal conductivity, which makes them better able to retain body heat and moisture. Unfortunately, polymer materials are synthetic and do not provide adequate air exchange between the skin and the environment.

An analysis of the market for specialized clothing for preterm infants to provide them with adequate biophysical comfort shows that the availability of clothing for preterm infants is very limited, with most clothing designed for babies weighing 2270–2700 g. Most of the products on offer are sleepwear with zippers or metal snaps made of cotton or polyester knits [13]. Clothing materials with enhanced physiological comfort parameters such as heat and moisture control are known. These materials, described in patent application CN 101792939, contain fibers with bamboo charcoal and Chinese herb extracts in their structure. The description provided indicates the ability of the patented garment construction to absorb moisture from the premature infant’s body rather than to reduce moisture loss [14].

According to the description of the patent application EP 2033614 A1, there is a three-layer structure intended for newborns or infants, in which one of the outer layers is an elastic layer intended for contact with the body, the second outer layer is a layer insulating the body, and the inner layer, enclosed between the outer layers, is a layer of liquid pumped in by pulsation, causing a change in pressure on particular regions of the body [15]. Only this solution can meet the goal of the present project, i.e., to obtain a garment for premature babies which is a barrier to the flow of heat and mass flux in the form of moisture. The barrier for heat flow is to protect the preterm baby from hypothermia and the barrier for mass transport is to protect from dehydration. Research on hospital bedding for newborns has been carried out for many years. The thermal resistance of hospital bedding is determined using thermal manikins of children [16,17].

In the literature, more attention is focused on the proper selection of raw materials for clothes for premature babies to eliminate irritating and allergic effects [18], as well as the proper design of clothes guaranteeing the child’s safety and manageability during medical procedures. The literature shows that seams should be flattened and delicate, all fasteners that facilitate dressing and undressing should be placed on the side or in the front of the product, and clothes should not contain tags and labels, as they may cause skin irritation. Garments for infants must not have cords, because there is a high probability of entangling cables from medical apparatus, or in other cases they may restrain the child’s body. Clothes must not have metal elements that can become hot and lead to burns. An important condition while designing clothes is functionality, connected with easy access to a newborn’s body, without the necessity of taking off the clothes in case of examination, connecting to medical apparatus, and in case of urgent medical procedures. Another aspect that in most cases determines the purchase of a given garment is aesthetics. Many parents attach more importance to the fact that the child, despite its small size, looks fashionable and attractive than to whether the product has been certified for safety [19].

Currently, preterm infants are dressed immediately after birth in a polyethylene bag called Calorkeeper by VYGON, which protects children from transdermal moisture loss. In accordance with the presented literature, there is an urgent need to develop technology for clothing for preterm infants which would simultaneously ensure maintenance of thermal balance and fluid mass from the body in physiological equilibrium, as well as being safe and easy to use, and would also look like children’s clothing.

Commercial knitted fabrics made of cotton and viscose were analyzed, with a thermal resistance of 0.018–0.032 m^2^·K·W^−1^ and vapor resistance of 4.5–9.5 m^2^·Pa·W^−1^. The thermal resistance for the VYGON product is at the level of 0.0057 m^2^·K·W^−1^ and the vapor resistance is above 900 m^2^·Pa·W^−1^. This high vapor resistance causes discomfort as a result of moisture on the skin and consequently cooling of the skin [20].

Therefore, the authors decided to develop materials that on one side maintain high vapor resistance to prevent evaporation of moisture from the body of a premature baby, and on the other side protect the baby from excessive moisture on the skin surface.

The best solution to achieve all the requirements of the appropriate biophysical comfort conditions would be to create a layered system consisting of different materials. The authors of this paper are working on the development of a three-layer system consisting of materials with conductive-diffusive properties on the skin side and sorptive properties on the outer side. The aim of this work is to select appropriate materials to develop protective garments that provide physiological comfort and safety to a premature baby based on layering systems that protect against heat and moisture loss. In order to protect prematurely born infants from the disturbance of the body heat balance, it is necessary to provide clothing with both defined thermal and vapor resistance.

## 2. Materials and Methods

### 2.1. Research Materials

The research material consisted of vapor-permeable foils, two-layer laminates, and knitwear differing mainly in surface mass. The vapor-permeable foils are made of polyethylene (PE) with different surface weights (Plastica, Kowalewo Pomorskie, Poland), two-layer laminates made of polypropylene (PP) non-woven fabric, and polyethylene (PE) foil with different surface weights (Plastica, Kowalewo Pomorskie, Poland), cotton (CO) and polyester (PES) knitwear (AMŁ Dzianiny, Lodz, Poland; Mirwal, Lodz, Poland) were tested. The composition of each material and its surface weights are shown in Table 1. In addition, samples were tested after the washing process. The washing process was carried out under conditions according to the guidelines for hospital laundering with a disinfectant for fabrics classified as medical devices (Ozonite BNL). The washing process was conducted at a temperature of 40 °C. Five washing cycles were performed. The washing time for 1 cycle was 20 min. After each washing cycle, rinsing was performed 5 times.

**Figure 1:**
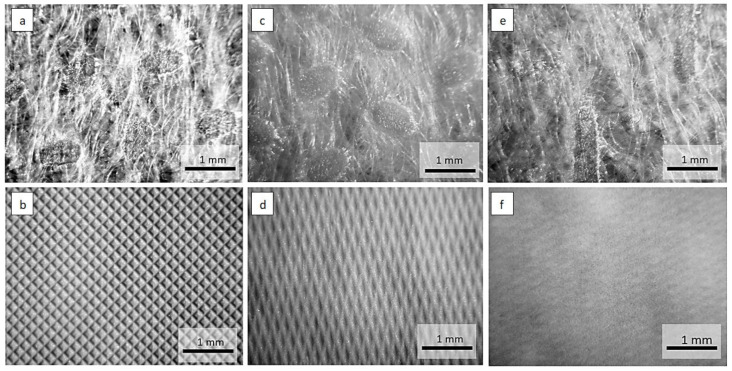
Optical microscope images of the materials: (**a**) non-woven side, (**b**) foil side of two-layer laminate (24 g·m^−2^ PE foil + 16 g·m^−2^ PP non-woven), (**c**) non-woven side, (**d**) foil side of two-layer laminate (30 g·m^−2^ PE foil + 35 g·m^−2^ PP non-woven), (**e**) non-oven side, (**f**) foil side of two-layer laminate (15 g·m^−2^ PE foil + 15 g·m^−2^ PP non-woven).

**Figure 2:**
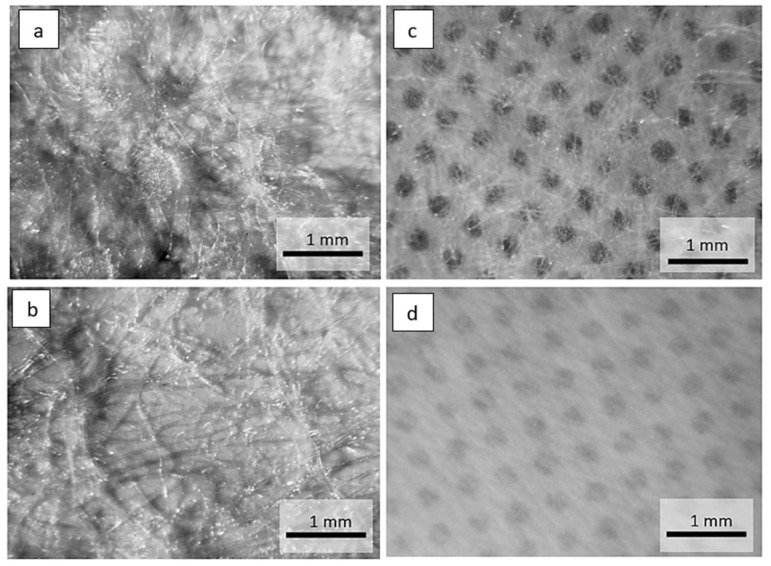
Optical microscope images of the materials: (**a**) non-woven side, (**b**) foil side of two-layer laminate (15 g·m^−2^ PE foil + 12 g·m^−2^ PP non-woven), (**c**) non-woven side, (**d**) foil side two-layer laminate (15 g·m^−2^ PE foil + 10 g·m^−2^ PP non-woven).

**Figure 3:**
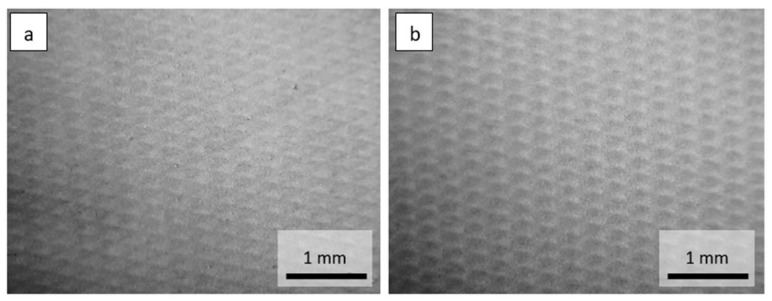
Optical microscope images of the materials: (**a**) Vapor-permeable PE foil 15 g·m^−2^, (**b**) Vapor-permeable PE foil 25 g·m^−2^.

**Figure 4:**
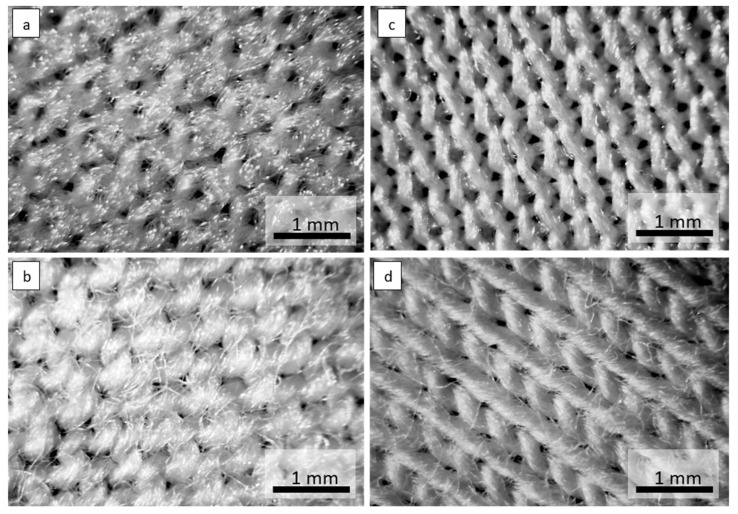
Optical microscope images of the materials: (**a**) knitwear interlock polyester 120 g·m^−2^, (**b**) knitwear single cotton 120 g·m^−2^, (**c**) knitwear interlock polyester 130 g·m^−2^, (**d**) knitwear single cotton 155 g·m^−2^.

Optical microscope images of the investigated materials are shown in Figure 1, Figure 2, Figure 3 and Figure 4.

Geometrically repeating non-woven bonding patterns are used to provide the non-woven web with isotropic dimensional stability. Heat or pressure is used to bond areas of the non-woven web, the non-woven is passed through a gap between heated calender rollers. The calender rollers may have different patterns of dimples and depressions on their surfaces. During the calendering process, the non-woven fibers are melted in the pattern areas. The microscopic images in Figure 1 and Figure 2 show cylindrical or elongated patterns on the non-woven side, which were created by the calendering process during laminate manufacturing.

### 2.2. Surface Mass Measurement

The surface mass test of the investigated materials was carried out according to the standard PN-EN12127 [21]. For the determination of the surface mass, 5 samples with a minimum surface area of 100 cm^−2^ were cut out. Measurements were taken at 3 locations along the length and 3 locations along the width of the specimen and the surface area of each specimen were calculated in this way.

### 2.3. Thickness Measurement

Measurement of the thickness of the tested materials was performed according to the standard PN-EN ISO 5084:1996 [22]. The thickness measurements were performed with a thickness gauge in 10 different places on the tested material sample. The result is the arithmetic mean of all measurements on a given sample.

### 2.4. Micro-Computed Tomography (Micro-CT)

A 3D reconstruction of tested laminates was made using X-ray micro-computed tomography (SkyScan 1272; Bruker, Kontich, Belgium). Micro-CT images of all tested laminates were obtained by applying the following scanning conditions: X-ray source voltage 50 kV, X-ray source current 200 µA, and pixel size 3.43 µm. A 180° rotation was performed with a rotation step of 0.2° and no filter was selected.

### 2.5. Measurement of Thermal Resistance and Vapor Resistance

The biophysical properties of the tested samples were evaluated in accordance with PN-EN ISO 11092:2014-11 [23]. The tests were performed using a textile evaluation station developed by Measurement Technology Northwest called Sweating Hotplate 8.2 in an air-conditioned chamber to ensure proper measurement conditions. The measurement of thermal resistance was performed at the plate temperature of 35 °C, the ambient temperature of 20 °C, and relative air humidity of 65%. The parameter related to the evaluation of the thermal resistance is calculated according to the following formula:

(1) $$R_{ct}=\frac{\left(T_{m}-T_{a}\right)\cdot A}{H-\Delta H_{c}}-R_{ct0}$$

where: *T_m_*—heating plate temperature [°C]; *T_a_*—air temperature [°C]; *A*—surface of the measuring plate [m^2^], *H*—heating power supplied to the measuring plate [W], ∆*H_c_*—heating power correction in case of measuring thermal resistance [W], *R_ct_*_0_—instrument constant for measuring thermal resistance, [m^2^·°C·W^−1^] and the parameter related to the assessment of water vapor resistance *R_et_*_0_ [m^2·^Pa·W^−1^] is calculated according to the formula:

(2) $$R_{et}=\frac{\left(p_{m}-p_{a}\right)\cdot A}{H-\Delta H_{e}}-R_{et0}$$

where: *p_m_*—saturated water vapor partial pressure [Pa] at the surface of the measuring plate at the temperature *T_m_*; *p_a_*—partial pressure [Pa] of water vapor in the air in the measuring chamber at the temperature *T_a_*; *A*—surface of the measuring plate [m^2^], *H*—heating power [W] supplied to the measuring plate; ∆*H_e_*—heating power correction [W] in the case of measuring the water vapor resistance, *R_et_*_0_—instrument constant [m^2^·Pa·W^−1^] for the measurement of water vapor resistance according to ISO 7243. The tests were carried out consecutively under the following conditions: thermal resistance: *T_a_* = 20 °C, RH = 65%, airflow speed 1 m·s^−1^; water vapor resistance: *T_a_* = 35 °C, RH = 40%, airflow speed 1 m·s^−1^.

### 2.6. Air Permeability Test

Tests of air permeability were carried out in accordance with PN-EN ISO 9237:1998 [24]. For measurements, Textest FX 3300-III device was used, which recorded the amount of air passing perpendicularly through the material; on the basis of these results, air permeability R of the material expressed in mm/s was calculated using Equation (3)

(3) $$R=\frac{\bar{q_{v}}}{A}\;\cdot \;167$$

where $\bar{q_{v}}$-arithmetic mean of the quantity of air flowing through the material [dm^3^·min^−1^]; *A*-surface area of tested product [cm^2^]; 167-factor converting liters per minute into square centimeters per second.

### 2.7. Kawabata Evaluation System

The Kawabata Evaluation System is a set of instruments used to measure the properties of textiles to predict the aesthetic values perceived by human touch. One group of these factors is the mechanical properties of flat textiles that determine sensory comfort. This comfort is determined objectively using a measurement system called Kawabata Evaluation System (KES). This system was used to evaluate the tested variants of flat textile products intended for infants’ garments at different stages of their development using modules that allow the analysis of the response of the material to varying loads such as:

– KES 1 examines shear and tensile forces
– KES 2 examines bending forces,
– KES 3 examines compressive forces,
– KES 4 examines surface properties (action of normal forces).

The KN-201-MDY equation, relating to textiles used in women’s closets for summer outfits, was chosen to determine the purpose of the materials tested, as it seemed most closely related to children’s garments, which should be soft.

In the apparel industry, the ‘hand’ is used as a measure of fabric quality and efficiency for a specific fabric application. The KES device enables the determination of fabric characteristics by automatically integrating the physical and surface properties of the fabric under study. The hand value that is determined using the KES device includes the basic hand value (HV) and the total hand value (THV). THV is a measure of tactile comfort, expressing the general sense of touch of the fabric, while HV provides details about the tactile properties of the fabric, such as stiffness, fullness, softness, and crispness (Koshi, Numeri, Fukurami). Stiffness (Koshi) is a feeling connected mainly with the rigidity of bending. This feeling is enhanced by the elastic properties of the fabric, i.e., by fabrics characterized by a high density, or fabrics made of elastic and resilient yarns. The sensation of smoothness (Numeri) is related to the sum of qualities defining smoothness, softness, and felting. Materials made from cashmere wool have a high value for this sensation. Fullness and softness (Fukurami) is a bulky, rich, and well-deformed feeling [25].

## 3. Results

**Figure 5:**
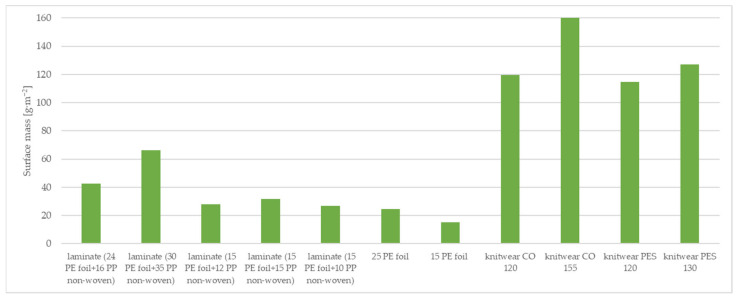
Surface mass of tested materials.

**Figure 6:**
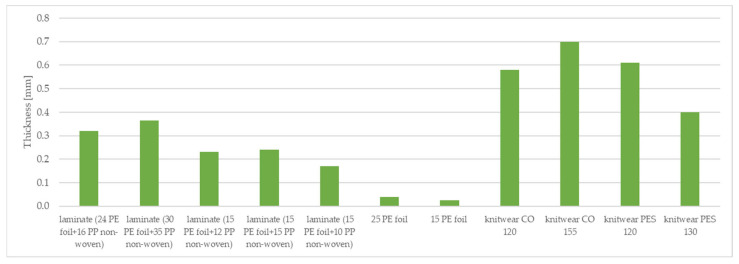
Thickness of tested materials.

The tested material samples were characterized in terms of surface mass and thickness. The results of the surface mass and thickness of the tested samples are shown in Table 2 and Figure 5 and Figure 6.

The surface mass and thickness of the materials affect the comfort of the garment. The materials investigated are components of the three-layer systems that will form the final garment materials. Clothing materials for premature babies should be sensitive and lightweight in order not to put additional load on the delicate skin of the premature baby. Therefore, it is essential to select materials characterized by low surface mass and thinness. Analyzing the results presented in Table 2, in the case of the tested two-layer laminate, the highest surface mass is that of two-layer laminate (30 g·m^−2^ PE foil + 35 g·m^−2^ PP non-woven), which is 66.09 g·m^−2^, while the lowest surface mass is that of two-layer laminate (15 g·m^−2^ PE foil + 10 g·m^−2^ PP non-woven), which is 26.78 g·m^−2^. Of the knitted fabrics, knitwear single cotton 155 g·m^−2^ had the lowest surface weight (160.81 g·m^−2^), while knitwear interlock polyester 120 g·m^−2^ had the lowest surface weight, which is 114.54 g·m^−2^. A vapor-permeable foil of 15 g·m^−2^ is characterized by the lowest surface mass and thickness of all the samples tested.

**Figure 7:**
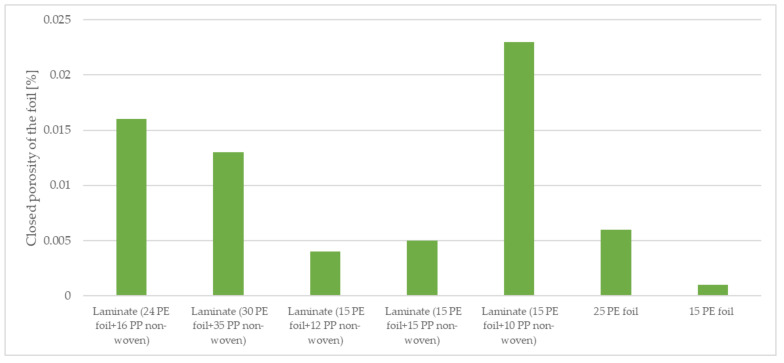
Closed porosity of the materials tested.

**Figure 8:**
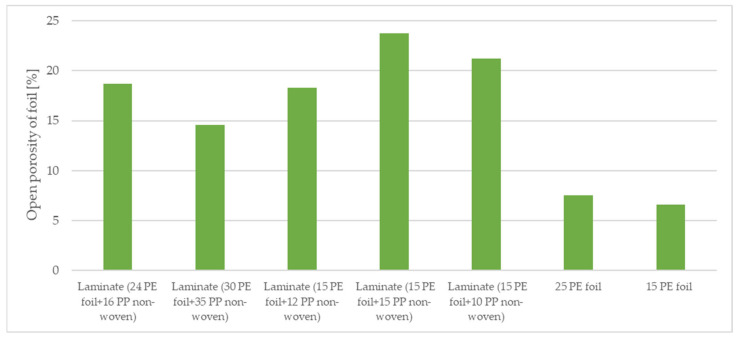
Open porosity of the tested materials.

The materials were subjected to computed microtomography to determine the porosity of the vapor-permeable membranes used to produce the three-layer systems. The porosity results are shown in Table 3 and Figure 7 and Figure 8.

**Figure 9:**
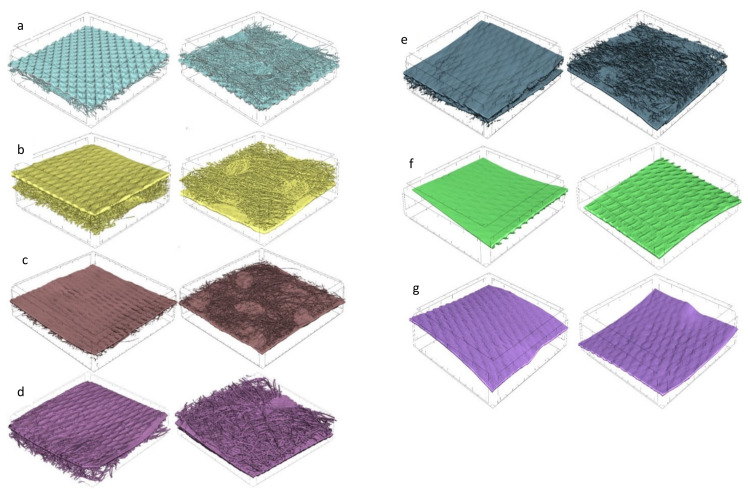
Micro-CT 3D reconstructions of tested textiles (all textiles have an area of 1 cm × 1 cm). (**a**) Two-layer laminate (24 g·m^−2^ PE foil + 16 g·m^−2^ PP non-woven); (**b**) Two-layer laminate (30 g·m^−2^ PE foil + 35 g·m^−2^ PP non-woven); (**c**) Two-layer laminate (15 g·m^−2^ PE foil + 12 g·m^−2^ PP non-woven); (**d**) Two-layer laminate (15 g·m^−2^ PE foil + 15 g·m^−2^ PP non-woven); (**e**) Two-layer laminate (15 g·m^−2^ PE foil + 10 g·m^−2^ PP non-woven); (**f**) Vapor-permeable PE foil 15 g·m^−2^; and (**g**) Vapor-permeable PE foil 15 g·m^−2^.

The foils were compressed after an additional bonding process with non-woven, which minimally affected their thickness. The open porosity (6.58%) and closed porosity (0.001%) of foil 15 g·m^−2^ are lower than that of open porosity (7.56%) and closed porosity (0.006%) of foil 25 g·m^−2^. The highest total porosity of foil was found in two-layer laminate (15 g·m^−2^ PE foil + 15 g·m^−2^ PP non-woven), which is 23.71%, and the lowest total porosity of foil was observed in two-layer laminate (30 g·m^−2^ PE foil + 35 g·m^−2^ PP non-woven), which is 14.59%. The ratio of open porosity of foil to closed porosity of foil represents about 1% of the closed pores. Images of the micro-CT materials investigated are illustrated in Figure 9.

The micro-CT 3D reconstructions also show the structures of the laminates from the non-woven side as well as the foil on the laminates and the structures of the foils. We can observe the places where the fibers were melted during the calendering process.

**Figure 10:**
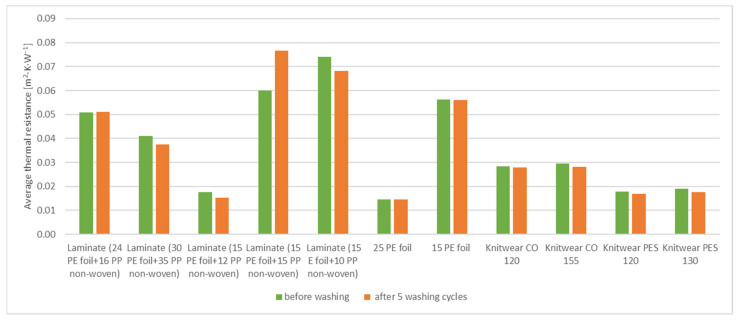
Average thermal resistance results: before and after 5 washing cycles.

**Figure 11:**
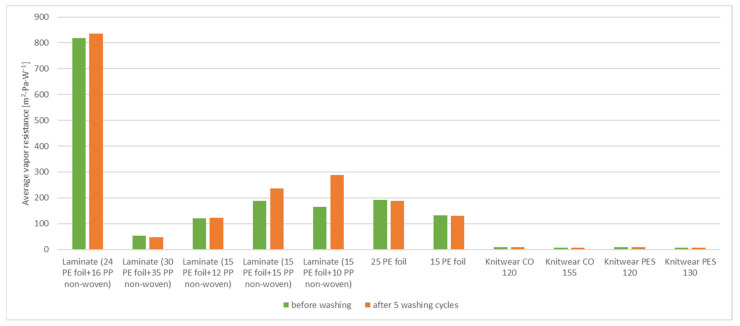
Average vapor resistance results: before and after 5 washing cycles.

This section contains the results of the biophysical properties and air permeability of the tested samples. Table 4 and Figure 10 and Figure 11 show the measured values of vapor resistance and heat resistance of the tested samples.

Analyzing the obtained data, it is observed that the sample of two-layer laminate (24 g·m^−2^ PE foil + 16 g·m^−2^ PP non-woven) is characterized by very high vapor resistance, which is at the level of 817.571 m^2^·Pa·W^−1^. The material with high vapor resistance can retain a large part of the evaporated vapor on the baby’s body surface, which can lead to overcooling of the preterm baby. The most optimal average thermal resistance was observed for two-layer laminate (15 g·m^−2^ PE foil + 15 g·m^−2^ PP non-woven) whose thermal resistance was 0.0766 m^2^·K·W^−1^, and two-layer laminate (15 g·m^−2^ PE foil + 10 g·m^−2^ PP non-woven), whose thermal resistance was 0.0683 m^2^·K·W^−1^. These samples also characterized the best vapor resistance results. For two-layer laminate (15 g·m^−2^ PE foil + 15 g·m^−2^ PP non-woven) vapor resistance was 188.729 m^2^·Pa·W^−1^ and for two-layer laminate (15 g·m^−2^ PE foil + 10 g·m^−2^ PP non-woven) vapor resistance was 164.085 m^2^·Pa·W^−1^. Vapor-permeable PE foil 25 g·m^−2^ also characterized optimal vapor resistance (192.176 m^2^·Pa·W^−^1). For knitted fabrics, the knitwear single cotton 155 g·m^−2^ showed the highest thermal resistance (0.0296 m^2^·K·W^−1^), and the knitwear interlock polyester 120 g·m^−2^ showed the lowest thermal resistance (0.0179 m^2^·K·W^−1^). The knitwear cotton 120 g·m^−2^ had the highest water vapor resistance (8.402 m^2^·Pa·W^−1^), while the knitwear interlock polyester 130 g·m^−2^ sample had the lowest resistance (6.356 m^2^·Pa·W^−1^). For a complete analysis, it is still necessary to carry out tests of three-layer systems, i.e., non-woven fabric/foil/knitted fabric systems.

The washing process slightly affected the results of the measurements of heat and vapor resistance of the tested materials. In some cases, the vapor resistance and the heat resistance of the tested samples increased, and in some cases, they decreased. The biggest difference before and after the washing process is observed in two-layer laminate (15 g·m^−2^ PE foil + 10 g·m^−2^ PP non-woven) and two-layer laminate (15 g·m^−2^ PE foil + 15 g·m^−2^ PP non-woven). The foil samples had the lowest differences in vapor resistance and heat resistance.

**Figure 12:**
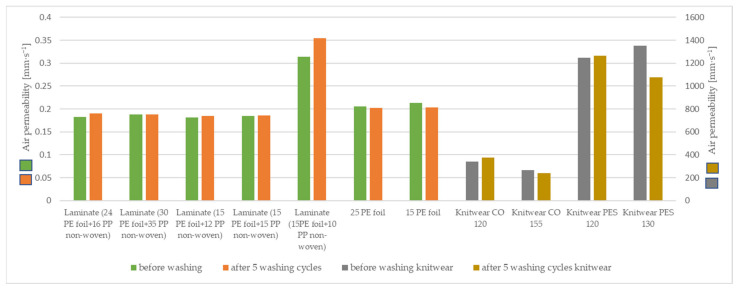
Air permeability of tested materials: before and after 5 washing cycles.

The air permeability of the tested materials is shown in Table 5 and Figure 12.

The laminates tested contained a non-woven fabric and a foil in their structure. The foil resists both vapor and heat, thus reducing air permeability. The two-layer laminate (15 g·m^−2^ PE foil + 10 g·m^−2^ PP non-woven) showed the highest air permeability (0.314 mm·s^−1^), while the two-layer laminate (15 g·m^−2^ PE foil + 12 g·m^−2^ PP non-woven) had the lowest air permeability (0.181 mm·s^−1^). The air permeability of the tested samples before and after washing did not change significantly, for some samples it remained the same. The air permeability of two-layer laminate (24 g·m^−2^ PE foil + 16 g·m^−2^ PP non-woven) and two-layer laminate (30 g·m^−2^ PE foil + 35 g·m^−2^ PP non-woven), two-layer laminate (15 g·m^−2^ PE foil + 12 g·m^−2^ PP non-woven) and two-layer laminate (15 g·m^−2^ PE foil + 15 g·m^−2^ PP non-woven) are at the same level. The only two-layer laminate (15 g·m^−2^ PE foil + 10 g·m^−2^ PP non-woven) had a different (higher) permeability. For knitted fabrics, the highest permeability was in the knitwear polyester interlock 130 g·m^−2^, while the lowest was in the knitwear single cotton 155 g·m^−2^. The biggest difference between the washed and unwashed knitwear sample was for the knitwear polyester interlock 130 g·m^−2^.

**Figure 13:**
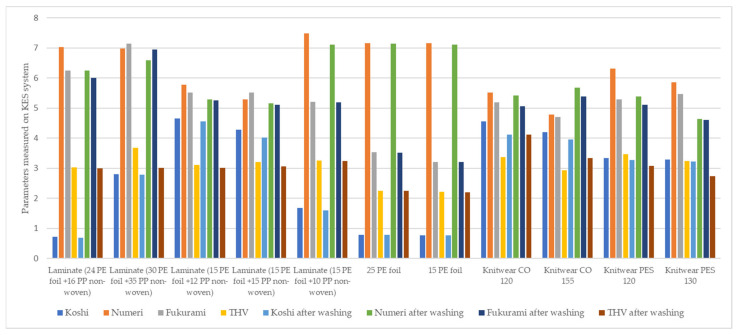
The parameters measured on KES system.

In order to characterize the mechanical properties of the materials used and to evaluate the total hand value, the samples were tested on the KES device. The results are included in Table 6 and Figure 13.

A bigger disparity was found in Koshi value with a maximum of 4.65 for two-layer laminate (15 g·m^−2^ PE foil + 12 g·m^−2^ PP non-woven) and a minimum of 0.72 for two-layer laminate (24 g·m^−2^ PE foil + 16 g·m^−2^ PP non-woven). Vapor-permeable PE foil 15 g·m^−2^ showed the worst fullness property with a value of 3.21 and two-layer laminate (30 g·m^−2^ PE foil + 35 g·m^−2^ PP non-woven) is found to be the bulkiest one. Numeri value of the knitwear single cotton 155 g·m^−2^ was the lowest (4.79), meaning that it was the harshest to touch, while the two-layer laminate (15 g·m^−2^ PE foil + 10 g·m^−2^ PP non-woven) showed the highest smoothness.

For knitted fabrics, knitwear single cotton 155 g·m^−2^ had the worst fullness and smoothness and the worst THV value. However, knitwear interlock polyester 120 g·m^−2^ had the highest numeri and THV values. Analyzing the tested laminates, the laminate (30 g·m^−2^ PE foil + 35 g·m^−2^ PP non-woven) had the highest Fukurami and THV parameters, while two-layer laminate (24 g·m^−2^ PE foil + 16 g·m^−2^ PP non-woven) had the lowest Koshi and THV values.

The Koshi, Numeri, Fukurami, and THV value results of the tested samples before and after washing did not change significantly, and for some samples, they remained the same.

**Figure 14:**
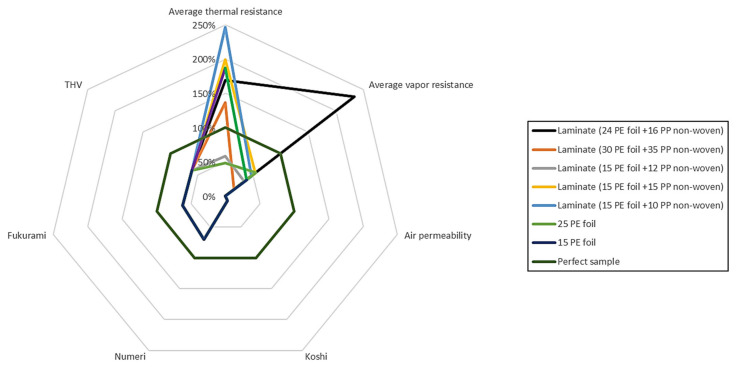
Sensory and biophysical properties of two-layer laminates and foils.

**Figure 15:**
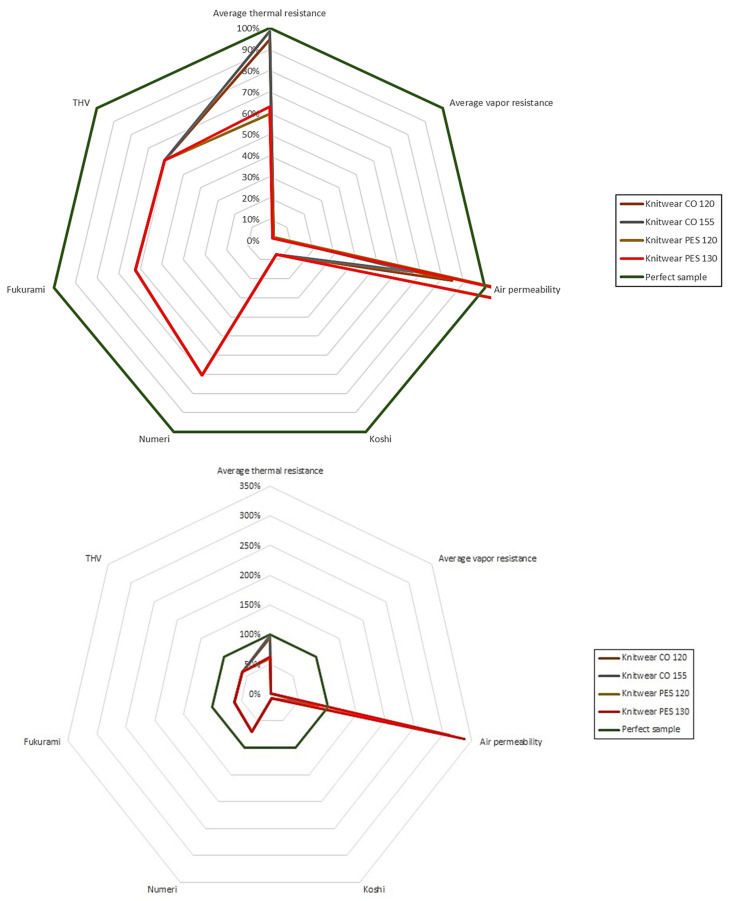
Sensory and biophysical properties of knitted fabrics.

Figure 14 and Figure 15 present the results of research on sensory and biophysical properties in order to better understand the problem related to the surface of textile products used on clothing for premature babies.

Figure 14 and Figure 15 show the relative percentage changes in the biophysical and sensory parameters of the tested materials. The ideal material was treated as a reference (green plot–regular octagon). It was assumed, based on the research of commercial materials and Vygon film, that the thermal resistance would be 0.03 m^2^·K·W^−1^, water vapor resistance −350 m^2^·Pa·W^−1^, and air permeability 400 mm·s^−1^. The best possible variants were adopted for the sensory comfort indicators (Numeri-10, Fukurami-10, Koshi-10, THV-5).

Analyzing the above data, it was observed that cotton knitted fabrics are the closest to the ideal among the knitted fabrics tested, while in the case of laminates, the closest to the ideal was two-layer laminate (15 g·m^−2^ PE foil + 15 g·m^−2^ PP non-woven).

## 4. Discussion

The aim of this study was to evaluate the most suitable materials for garments for prematurely born infants in order to find the best items that would fulfill their protective function. The purpose of protective clothing is to prevent excessive loss of body heat and moisture from premature infants, which can lead to hypothermia. In order to provide a suitable environment for the development of a premature baby, the designed garment should have, besides sensory characteristics, suitable biophysical characteristics influencing the biophysical comfort of the infant. Clothing systems should provide adequate insulation of heat and moisture in such a way that water and heat are not lost through the body. In addition, the material of the premature infant’s garment is also an important consideration.

The highest thermal resistance was characteristic for the samples: two-layer laminate (15 g·m^−2^ PE foil + 10 g·m^−2^ PP non-woven), which was 0.0740 m^2^·K·W^−1,^ and two-layer laminate (15 g·m^−2^ PE foil + 15 g·m^−2^ PP non-woven), which was 0.0599 m^2^·K·W^−1^.

The vapor-permeable foil 15 g·m^−2^ also obtained optimal results (0.0562 m^2^·K·W^−1^). Therefore, it can be concluded, on the basis of the performed tests, which sets of those materials best maintain the constant temperature between the environment and the child’s skin during the flow of a dry heat stream.

On the other hand, analyzing the vapor resistance, the most optimal parameters were characterized by vapor-permeable foil 25 g·m^−2^ and two-layer laminate (15 g·m^−2^ PE foil + 15 g·m^−2^ PP non-woven) and two-layer laminate (15 g·m^−2^ PE foil + 10 g·m^−2^ PP non-woven).

The surface weight of the materials is also an important parameter in the selection of materials for garments for premature babies. Samples that have shown the best vapor resistance and heat resistance properties have a surface weight of 27–32 g·m^−2^. Of the laminates tested, the two-layer laminate (30 g·m^−2^ PE foil + 35 g·m^−2^ PP non-woven) has the highest surface mass and the two-layer laminate (15 g·m^−2^ PE foil + 10 g·m^−2^ PP non-woven) has the lowest surface mass. Of all the samples tested, the lowest surface mass is characterized by the vapor-permeable foil 15 g·m^−2^.

According to the study of the conducted tests of laminates, vapor-permeable foil, and knitwear, it can be concluded that the most optimal materials for clothing for premature babies are vapor-permeable two-layer laminate (15 g·m^−2^ PE foil + 15 g·m^−2^ PP non-woven) and vapor-permeable two-layer laminate (15 g·m^−2^ PE foil + 10 g·m^−2^ PP non-woven).

Total Hand Value (THV) for cotton knitted fabrics improved after the washing process, while THV value for polyester knitted fabrics deteriorated. Considering this fact, cotton knitwear will be selected for further study. Considering the surface mass of the knitted fabric also, which should be as low as possible for premature baby clothes, the best knitted fabric is knitwear single cotton 120 g·m^−2^.

The next step is to choose the right knitwear to create a three-layer system, which will be the final and most optimal clothing system for premature babies.

## 5. Conclusions

The presented paper was aimed at evaluating the biophysical properties of selected materials or layered systems, which will be a part of a three-layer system from which a protective garment for prematurely born children will be made. In order to maintain the biophysical comfort of the premature baby, it is necessary to select appropriate materials for garments, the parameters of which will fulfill the requirements. Protective garments for premature babies should act as a heat and moisture insulator. The clothing has to protect the preterm neonate from excessive water and heat loss from the body which can lead to hypothermia. The garment design for premature babies should be characterized by moisture barrier properties and high thermal insulation. It should also have optimal air permeability properties. The main barrier to heat and water vapor is the vapor-permeable film from which laminates are constructed, so in the case of laminates, the most important parameters to consider when choosing the best materials are water vapor and heat resistance, and air permeability. Sample two-layer laminate (15 g·m^−2^ PE foil + 15 g·m^−2^ PP non-woven) had the best water vapor resistance and thermal insulation properties. Moreover, this sample was characterized by better air permeability and surface weight than the other samples of laminates. When designing garments for premature babies it is important to reduce the surface weight to as low as possible as the skin of a premature baby does not have a stratum corneum structure and should not be burdened. Among the knitted fabrics, a knitwear single cotton 120 g·m^−2^ knitwear polyester interlock 120 g·m^−2^ was selected for having the best THV or tactile comfort. In addition, these knits were chosen for their lower surface weight.

Clothing fabrics for premature babies should be soft, delicate, and smooth. Stiff materials with a high Koshi value are not suitable for premature babies due to their immature, delicate and irritable skin. The lower the Koshi value, the harsher the fabric, which is not desirable for premature babies’ garments. The materials selected for further testing are characterized by a fairly high bending stiffness of the selected materials because they contain elastic raw materials with a high density. The results of the selected materials are at the desired level. Clothes for premature babies should be smooth and soft, therefore the higher the Fukurami and Numeri value, the better.

Based on the conducted tests, two-layer laminate (15 g·m^−2^ PE foil + 15 g·m^−2^ PP non-woven), the knitwear single cotton 120 g·m^−2,^ and knitwear polyester interlock 120 g·m^−2^ were selected for further research.

The experimental part of the study included the investigation of surface mass, material thickness, as well as air permeability, thermal resistance, and vapor resistance of the tested materials, and mechanical properties (Koshi, Numeri, Fukurami, and THV). Moreover, optical microscope images and CT scan images were taken to characterize the surface of the samples. The materials were subjected to micro-CT to determine the porosity of the vapor-permeable membranes used to produce the garments for preterm babies.

During the design of protective clothing for premature babies, materials with moisture barrier properties should be used, which should be characterized by high thermal insulation and at the same time ensure appropriate comfort of use.

Prematurity is associated with a number of risks for the newborn, the most common of which include the cooling down of the body—premature babies have a low weight, too little fat tissue, and poorly developed muscles, therefore they cannot maintain a constant body temperature. Therefore, they are placed in a special, foil-covered cot with a radiant heater in order to minimize the loss of heat and fluids through the skin or incubator.

In order to adapt a premature baby to proper functioning, it is necessary to maintain a constant body temperature. Thermal equilibrium is the result of the amount of heat generated and lost by the newborn and the amount of heat supplied to the newborn from the outside. The condition for maintaining the thermal equilibrium is the proper functioning of the mechanisms responsible for the feeling of heat and cold and the occurrence of a fully developed response to the changing thermal conditions of the environment. The immature autonomic system of premature babies causes a much slower and weaker response to cooling, making this group of patients particularly vulnerable to the dangerous consequences of thermoregulation disorders. The hypothalamus plays an important role in thermoregulation processes. When the temperature limit of 37 °C is exceeded in the hypothalamus, the processes of sweating and expansion of peripheral vessels are activated in order to cool the body down. On the contrary, if the temperature drops, the peripheral vessels constrict and catecholamine production increases. Heat loss is especially acute in premature babies and occurs as a result of the evaporation of amniotic fluid, the evaporation of water left on the baby’s skin, or the evaporation of cosmetic or medicinal substances that cover the body of the newborn.

In the first days of their lives, newborns with a very low body weight (<1500 g) lose heat mainly through evaporation, multiplied by thin, immature skin, that is poor in a protective keratin layer, and extremely water-permeable. The amount of water lost in this way is eight times higher than that of adults. The technology being developed is aimed at eliminating the negative factors associated with body cooling and excessive loss of physiological fluids from the child’s body.

## Figures and Tables

**Table 1:** Characterization of the materials.

Materials	Surface Mass/Composition		Abbreviation
	Foil	Non-Woven/Knitwear	
Two-layer laminate	24 g·m^−2^, PE	16 g·m^−2^, PP	Laminate (24 PE foil +16 PP non-woven)
Two-layer laminate	30 g·m^−2^, PE	35 g·m^−2^, PP	Laminate (30 PE foil +35 PP non-woven)
Two-layer laminate	15 g·m^−2^, PE	12 g·m^−2^, PP	Laminate (15 PE foil +12 PP non-woven)
Two-layer laminate	15 g·m^−2^, PE	15 g·m^−2^, PP	Laminate (15 PE foil +15 PP non-woven)
Two-layer laminate	15 g·m^−2^, PE	10 g·m^−2^, PP	Laminate (15 PE foil +10 PP non-woven)
Vapor-permeable foil	25 g·m^−2^, PE	-	25 PE foil
Vapor-permeable foil	15 g·m^−2^, PE	-	15 PE foil
Knitwear single cotton	-	120 g·m^−2^, CO	Knitwear CO 120
Knitwear single cotton	-	155 g·m^−2^, CO	Knitwear CO 155
Knitwear interlock polyester	-	120 g·m^−2^, PES	Knitwear PES 120
Knitwear interlock polyester	-	130 g·m^−2^, PES	Knitwear PES 130

**Table 2:** Surface mass and thickness of materials.

Materials	Surface Mass [g·m^−2^]	Thickness [mm]
Laminate (24 PE foil +16 PP non-woven)	42.46 ± 1.03	0.32 ± 0.02
Laminate (30 PE foil +35 PP non-woven)	66.09 ± 0.53	0.36 ± 0.02
Laminate (15 PE foil +12 PP non-woven)	27.93 ± 0.28	0.23 ± 0.01
Laminate (15 PE foil +15 PP non-woven)	31.61 ± 0.20	0.24 ± 0.02
Laminate (15 PE foil +10 PP non-woven)	26.78 ± 0.28	0.17 ± 0.01
25 PE foil	24.49 ± 0.67	0.04 ± 0.01
15 PE foil	15.23 ± 0.25	0.03 ± 0.01
Knitwear CO 120	119.74 ± 1.39	0.58 ± 0.02
Knitwear CO 155	160.81 ± 4.20	0.7 ± 0.01
Knitwear PES 120	114.54 ± 0.89	0.61 ± 0.02
Knitwear PES 130	127.24 ± 4.21	0.4 ± 0.01

**Table 3:** Results from micro-CT test—porosity of vapor-permeable membranes.

Materials	Thickness of Laminate [μm]	Thickness of Foil [μm]	Thickness of Non-Woven [μm]	Total Porosity of the Foil [%]	Closed Porosity of the Foil [%]	Open Porosity of the Foil [%]
Laminate (24 PE foil +16 PP non-woven)	499	98	401	18.67	0.016	18.66
Laminate (30 PE foil +35 PP non-woven)	685	102	583	14.59	0.013	14.58
Laminate (15 PE foil +12 PP non-woven)	390	72	318	18.30	0.004	18.29
Laminate (15 PE foil +15 PP non-woven)	451	73	378	23.71	0.005	23.71
Laminate (15 PE foil +10 PP non-woven)	284	71	213	21.23	0.023	21.23
25 PE foil	101	101	-	7.56	0.006	7.56
15 PE foil	78	78	-	6.58	0.001	6.58

**Table 4:** The results of vapor resistance and heat resistance obtained for the tested materials.

Materials	Average Thermal Resistance [m^2^·K·W^−1^]	Average Thermal Resistance after 5 Washing Cycles [m^2^·K·W^−1^]	Average Vapor Resistance [m^2^·Pa·W^−1^]	Average Vapor Resistance after 5 Washing Cycles [m^2^·Pa·W^−1^]
Laminate (24 PE foil +16 PP non-woven)	0.0508 ± 0.0002	0.0511 ± 0.0002	817.571 ± 7.679	834.937 ± 7.987
Laminate (30 PE foil +35 PP non-woven)	0.0410 ± 0.0003	0.0376 ± 0.0003	54.209 ± 1.897	48.015 ± 1.754
Laminate (15 PE foil +12 PP non-woven)	0.0176 ± 0.0008	0.0153 ± 0.0008	120.209 ± 3.977	122.033 ± 4.024
Laminate (15 PE foil +15 PP non-woven)	0.0599 ± 0.0015	0.0766 ± 0.0015	188.729 ± 1.2497	235.817 ± 3.013
Laminate (15 PE foil +10 PP non-woven)	0.0740 ± 0.002	0.0683 ± 0.002	164.085 ± 1.358	289.142 ± 1.858
25 PE foil	0.0145 ± 0.001	0.0145 ± 0.001	192.176 ± 4.673	188.988 ± 4.572
15 PE foil	0.0562 ± 0.0007	0.0561 ± 0.005	132.398 ± 1.3937	130.125 ± 1.206
Knitwear CO 120	0.0284 ± 0.002	0.0279 ± 0.003	8.402 ± 0.162	8.396 ± 0.123
Knitwear CO 155	0.0296 ± 0.001	0.0281 ± 0.001	7.863 ± 0.095	7.845 ± 0.092
Knitwear PES 120	0.0179 ± 0.003	0.0169 ± 0.002	8.292 ± 0.150	8.257 ± 0.136
Knitwear PES 130	0.0189 ± 0.008	0.0176 ± 0.005	6.356 ± 0.067	6.346 ± 0.069

**Table 5:** Air permeability of tested materials.

Materials	Air Permeability [mm·s^−1^]	Air Permeability after 5 Washing Cycles [mm·s^−1^]
Laminate (24 PE foil +16 PP non-woven)	0.183 ± 0.004	0.190 ± 0.005
Laminate (30 PE foil +35 PP non-woven)	0.188 ± 0.003	0.188 ± 0.006
Laminate (15 PE foil +12 PP non-woven)	0.181 ± 0.004	0.184 ± 0.005
Laminate (15 PE foil +15 PP non-woven)	0.185 ± 0.008	0.185 ± 0.005
Laminate (15 PE foil +10 PP non-woven)	0.314 ± 0.020	0.355 ± 0.006
25 PE foil	0.206 ± 0.009	0.202 ± 0.010
15 PE foil	0.213 ± 0.019	0.204 ± 0.016
Knitwear CO 120	338.4 ± 16.1	376.6 ± 8.6
Knitwear CO 155	264.4 ± 15.4	237.6 ± 13.7
Knitwear PES 120	1246 ± 20.7	1266 ± 25.9
Knitwear PES 130	1350 ± 18.7	1178 ± 21.5

**Table 6:** The parameters measured on KES device.

Materials		Before Washing				After 5 Washing Cycles		
	Koshi	Numeri	Fukurami	THV	Koshi	Numeri	Fukurami	THV
Laminate (24 PE foil +16 PP non-woven)	0.72	7.03	6.24	3.03	0.69	6.25	6.01	2.99
Laminate (30 PE foil +35 PP non-woven)	2.79	6.97	7.14	3.67	2.78	6.58	6.95	3.01
Laminate (15 PE foil +12 PP non-woven)	4.65	5.78	5.52	3.10	4.55	5.28	5.26	3.01
Laminate (15 PE foil +15 PP non-woven)	4.27	5.28	5.52	3.21	4.02	5.15	5.11	3.06
Laminate (15 PE foil +10 PP non-woven)	1.67	7.48	5.21	3.25	1.59	7.11	5.19	3.24
25 PE foil	0.78	7.15	3.53	2.25	0.78	7.14	3.52	2.25
15 PE foil	0.76	7.16	3.21	2.21	0.76	7.11	3.20	2.20
Knitwear CO 120	4.55	5.51	5.19	3.36	4.12	5.42	5.06	4.11
Knitwear CO 155	4.19	4.79	4.70	2.93	3.96	5.68	5.39	3.34
Knitwear PES 120	3.33	6.31	5.29	3.47	3.27	5.39	5.10	3.07
Knitwear PES 130	3.29	5.85	5.46	3.24	3.22	4.64	4.60	2.73

## Data Availability

Not applicable.
